# Evaluation of the Mistakes in Self-Diagnosis of Sexual Dysfunctions in 11,000 Male Outpatients: A Real-Life Study in An Andrology Clinic

**DOI:** 10.3390/jcm8101679

**Published:** 2019-10-14

**Authors:** Giovanni Burgio, Bruno Giammusso, Aldo E. Calogero, Daniele Mollaioli, Rosita A. Condorelli, Emmanuele A. Jannini, Sandro La Vignera

**Affiliations:** 1 Department of Clinical and Experimental Medicine, University of Catania, 95125 Catania, Italy; burgio.giovanni88@libero.it (G.B.); acaloger@unict.it (A.E.C.); rosita.condorelli@unict.it (R.A.C.); 2 Urology Clinic, Policlinic “Morgagni”, 95125 Catania, Italy; bgiammusso@hotmail.it; 3 Chair of Endocrinology and Medical Sexology (ENDOSEX), Department of Systems Medicine, University of Rome Tor Vergata, 00133 Rome, Italy; daniele.mollaioli@gmail.com (D.M.); eajannini@gmail.com (E.A.J.)

**Keywords:** erectile dysfunction, premature ejaculation, hypoactive sexual desire disorder, unmet needs

## Abstract

Purpose: The aim of this study was to compare the initial request for sexual consultation with the final diagnosis and to evaluate the limits of the active andrological anamnesis concerning unclassified male sexual dysfunction. Methods: In this 12-year observational retrospective study, we collected data from patients referring to an andrological outpatient clinic, evaluating the requests, perceptions, needs, and self-diagnosis at their first visit and comparing them with the final diagnosis reached after a complete clinical, laboratory, and instrumental investigation. Results: A total of 11,200 patients were evaluated. The main request of andrological consultation was erectile dysfunction (ED) (52%), followed by premature ejaculation (PE) (28%), and low sexual desire (11.5%). Among the patients seeking help for ED, about 30% were ultimately found to have a different type of dysfunction and 24% were diagnosed with an “unmet need”, which included issues not present in the current nosography nonetheless affecting sexual and relational life. Among the patients referring for PE, the final diagnosis was lifelong PE for the large majority of them, regardless of whether initially they thought to have an acquired form. Several of those who sought consultation for acquired PE were frequently found to be able to compensate for lifelong PE by a subsequent coitus or were able to induce orgasm in the partner with different modalities. Among the patients referring for low sexual desire, only 57.5% were confirmed to have it; 23% had ED and 18.5% showed a raised threshold of penile sensitivity. Conclusions: The results of this study show that the reason for consultation is frequently misleading and raise the relevance of being aware of the so-called “unmet needs” and to discuss with the patient and the couple to explore the sexual history behind the self-diagnosis. These findings also suggest the need to expand the current taxonomy of male sexual dysfunctions.

## 1. Introduction

The most frequent sexual complaints of male patients are related to erection, ejaculation, and desire. Erectile dysfunction (ED) is a common male sexual dysfunction that may be due to a dysfunction of any component of the erectile function, including organic (vasculogenic, endocrine, anatomical neurogenic, and iatrogenic) factors, and non-organic (relational and intrapsychic) factors [1]. Two milestone studies have risen the awareness of its epidemiology: the Massachusetts Male Ageing Study (MMAS) [2] and the European Male Ageing Study (EMAS) [3]. The MMAS showed that the combined prevalence of mild–moderate ED was 52% in men aged 40–70 years; ED was strongly related to age, health status, and emotional function. Conversely, the EMAS, the largest European multicenter population-based study of ageing men (40–79 years), reported a prevalence of ED ranging from 6% to 64%, depending on different age subgroups. Prevalence increased with age, with an average prevalence of 30%. The prevalence of ED in younger men has been poorly highlighted [4,5]. In this context, a study reported that one man out of four who seeks medical help for ED in the real-life setting is <40 years of age [6]. Another study showed that 22.1% of men <40 years of age had low (<21) Sexual Health Inventory for Men (SHIM) scores [7].

The definition of premature ejaculation (PE) has changed over the years. An expert panel of the International Society of Sexual Medicine experts proposed, in 2007, and again in 2013, a unified definition of both lifelong and acquired PE: (i) ejaculation that always or nearly always occurs prior to or within about one minute of vaginal penetration from the first sexual experience (LLPE), or a clinically significant decrease in latency time, often to about 3 min or less (acquired PE, APE); (ii) the inability to delay ejaculation on all or nearly all vaginal penetrations; and (iii) negative personal consequences, such as distress, bother, frustration, and/or the avoidance of sexual intimacy [8,9,10]. An approximately 5% prevalence of LLPE and APE in the general population is consistent with epidemiological data indicating that around 5% of the population has an ejaculation latency of less than 2 min [9]. Moreover, ejaculatory dysfunctions (EjDs) other than PE are a spectrum of distressful ejaculatory symptoms that include delayed ejaculation (DE), anejaculation (AE), perceived ejaculate volume reduction (PEVR), or decreased force of ejaculation (DFE) [11]. In addition, in this case, several organic and nonorganic risk factors have been described, but their prevalence in the the general population is relatively poorly assessed [12].

Libido, or sexual drive, is a psychosomatic function that can be affected by various factors that include organic ones (hormonal derangements or systemic diseases), life-style related (alcoholism or drug abuse), intrapsychic (depression and other psychiatric diseases), or environmental (relational issues or distress) [13,14,15,16]. Longitudinal studies have found that libido declines with increasing male age [14]. When assessing libido, many studies use the sexual desire (SD) domain of the International Index of Erectile Dysfunction (IIEF), which asks two libido-related questions: “Over the past 4 weeks, how often have you felt sexual desire?” and “Over the past 4 weeks, how would you rate your level of sexual desire?” The IIEF-SD questions can be used to diagnose mild, mild to moderate, moderate, or severe dysfunction [17]. Other studies have used their own scale, such as the Sexual Arousal, Interest and Drive scale (SAID)—a validated patient reported outcomes measuring five scored items, including sexual thought, arousal, as well as interest and drive [18], or some others [16].

Since the introduction of sildenafil and in recent years, the awareness of male sexual health has raised up; thus, an increasing number of patients seek andrological consultation [19,20]. The need for a correct interpretation of the anamnestic data is of paramount importance, since the patient often has difficulties in describing his innermost sexual experience, thus misleading the clinician from the real occurring condition. Although sexology has been defined in an evolving way and includes different conditions within the spectrum of the male sexuality, many situations described by male patients are not well classified, since their presentation is subtle, not clearly perceived, difficult or embarrassing to describe, or the real problem may show up after a phase of behavioral compensation. Thus, clinical and scientific communities are called to constantly update not only the definitions and criteria of the existing sexual dysfunctions, but also to identify new taxonomic categories for not classified or sub-clinical sexual conditions [21,22]. In this 12-years long observational study, we collected data from a large cohort of male patients, evaluating the initial request for consultation and the final diagnosis, enlightening the differences found between the sexual issues perceived and referred by the patients at the time of their first outpatient consultation and the real conditions found after a complete diagnostic work-up.

## 2. Patients and Methods

Study approval was granted by the Intradivisional Ethical Committee and a written informed consent was obtained from each patient (Code: 1/2011). Data from male patients referring to the Andrological clinic (University Teaching Hospital, University of Catania, Italy) were collected from May 2011 to December 2018. The requests for the andrological consultation were collected and classified as follows: ED, PE divided in LLPE and APE, low sexual desire (either due to a relational problem—in the majority of cases—or rarely a real hypoactive sexual desire disorder caused by endocrinological or psychiatric issues—HSDD), DE, and a number of other symptoms that include other EjDs and dysmorphophobia. Furthermore, a subgroup of complains were recorded and classified as “unmet needs”. They consisted of situations of uncertain presentation that, although not classified among the classical sexual dysfunctions, cause distress in the male’s or couple’s sexual life [21]. The same classification was used for the final diagnosis, obtained after a thorough anamnestic, laboratory and instrumental evaluation, as described below.

Erectile function was assessed using the abridged International Index of Erectile Function (IIEF-5): an IIEF-5 score <21 was used to define ED in the present study.

PE was defined according to the ISSM definition and PE severity was assessed using the Premature Ejaculation Diagnostic Tool (PEDT), a five-item unidimensional measure which captures the major aspects of ejaculation status diagnosis: control, frequency, minimal stimulation, distress, and interpersonal difficulty. A score of ≤8 indicated no-PE, 9 and 10 probable PE, and ≥11 PE [23]. Onset was defined as lifelong if the patient reported that it had occurred since the first sexual intercourse or acquired if it started later during sexual life.

After questionnaire administration, all patients underwent a biochemical evaluation (measurements total testosterone, prolactin, TSH, glycemia, total cholesterol, HDL, triglycerides, and uricemia; when phenotypic or familiar factors suggested hyperinsulinemia or insulin-resistance, evaluation of insulin was added). Penile doppler dynamic ultrasound with prostaglandin stimulation, as well other laboratory and instrumental evaluations, were used according to the peculiar clinical history of each subject. If patients had neither altered scores at questionnaires, nor biochemical or vascular alterations, they were included in the “unmet needs” group and further sorted through a second non-questionnaire-based interview.

Data were reported as the prevalence and percentage of patients affected by the previously listed conditions.

## 3. Results

A total of 11,200 male patients aged from 15 to 91 years (mean age 51.7 years) were evaluated during the observational period. The most frequent reason of consultation was ED (52%), followed by PE (28% total; 9% for LLPE, 19% for APE), low sexual desire (11.5%) and DE or other EjDs (4%). A low percentage of patients (4%) referred more heterogeneous symptoms. These included “unmet needs” such as lower maximal penile rigidity and/or soft glans during penetration (subclinical ED, SED) [21] that, by definition, did not preclude the sexual intercourse, prolonged the refractory period, increased the threshold to stimuli (an increase of the necessary psychosomatic stimuli needed to induce penile rigidity, compared to a previous situation), a decreased number of spontaneous, nocturnal, and morning erections, delayed or absent orgasm in the female partner secondary to any condition in the male partner (Table 1).

The diagnoses achieved after careful clinical and instrumental investigation were frequently different. The most common final diagnosis was ED (40%), followed by LLPE (24.9%), “unmet needs” (23.9%), low sexual desire, mostly linked to relational issues (8.6%), and APE (1.6%) (Table 2).

**Figure 1:**
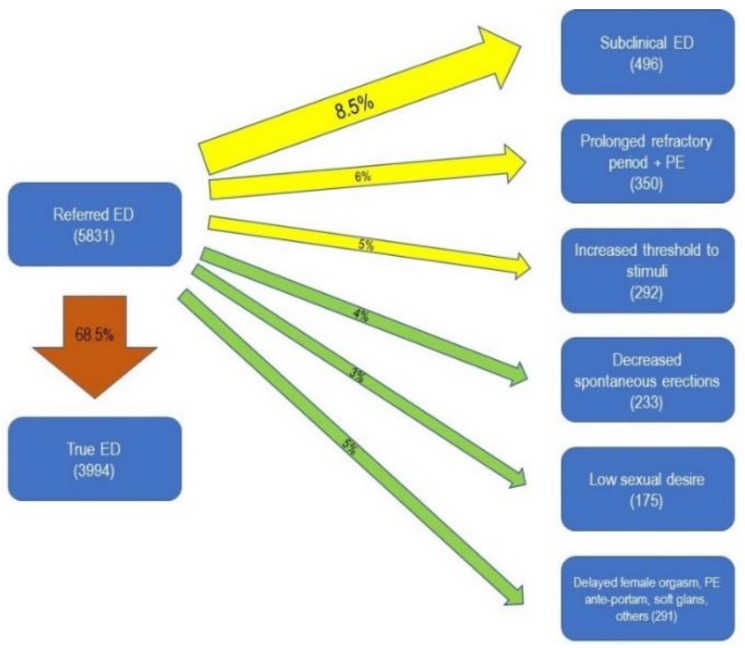
Prevalence of final diagnoses in patients who reported erectile dysfunction (ED) at the first visit to the andrology clinic. ED: Erectile Dysfunction; PE: premature ejaculation.

Concerning the patients who sought consultation for ED, the final diagnoses were: true ED found in more than two thirds of the patients (68.5%), whereas the rest were diagnosed with subclinical ED (8.5%), a prolonged refractory period plus PE (6%), an increased threshold to stimuli (5%), decreased spontaneous erections (4%), low sexual desire (3%), delayed female orgasm (1.5%), ejaculation before penetration (PE ante-portas) (0.7%), and other conditions (2.5%) (Figure 1).

**Figure 2:**
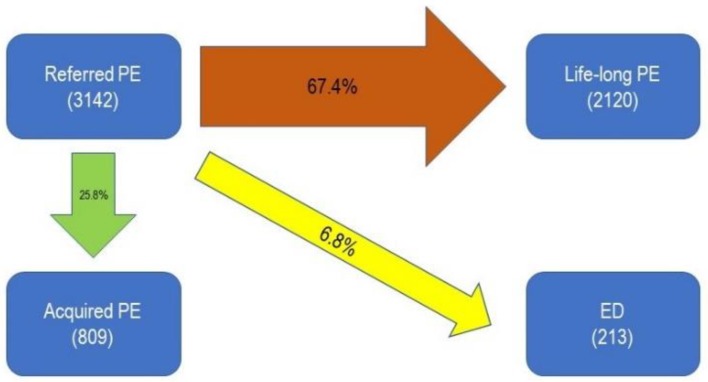
Prevalence of final diagnoses in patients who reported premature ejaculation (PE) at the first visit to the andrology clinic. ED: Erectile Dysfunction; PE: premature ejaculation.

The majority of patients claiming PE as a symptom for consultation were found, at the final diagnosis, to have LLPE (67.4%), while 25.8% had true APE and 6.8% ED without PE (Figure 2).

**Figure 3:**
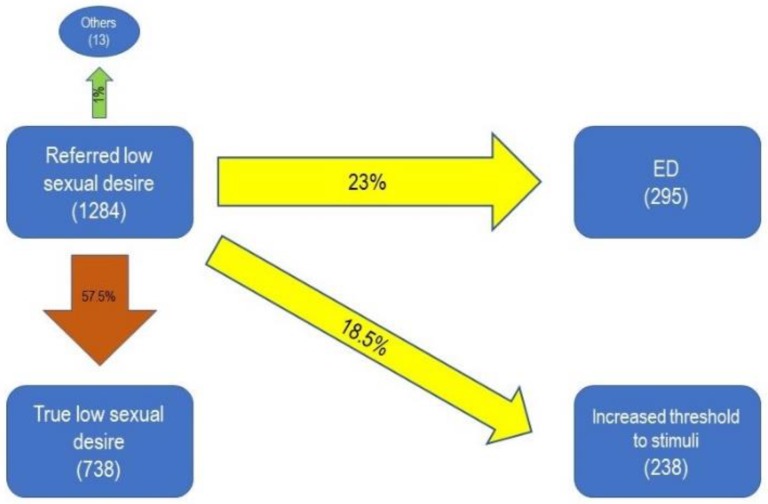
Prevalence of final diagnoses in patients who complained of hypoactive sexual desire disorder (HSDD) at the first visit to the andrology clinic. ED: Erectile Dysfunction; HSSD: Hypoactive Sexual Desire Disorder; Others: delayed female orgasm, prolonged refractory period and PE, decreased spontaneous erections, soft glans.

The patients referring low sexual desire as the first complaint were diagnosed as having a real, primitive, and absolute libido problem only in about half of the cases (57.5%), whereas the rest were found to have ED (23%), increased threshold to stimuli (18.5%), or other conditions (1%) (Figure 3).

**Figure 4:**
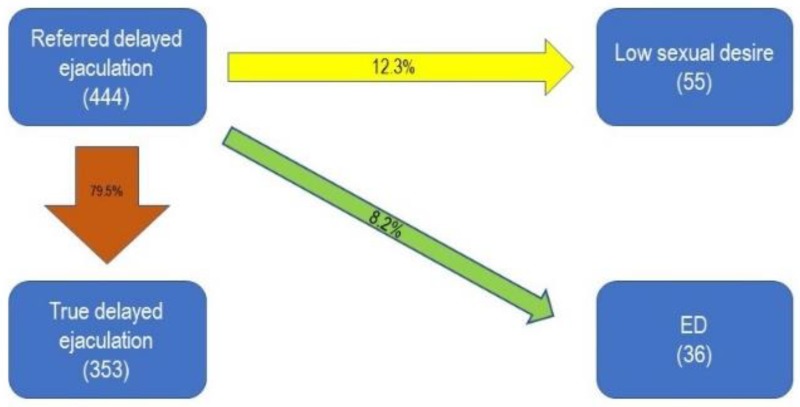
Prevalence of final diagnoses in patients who complained of delayed ejaculation at the first visit to the andrology clinic. ED: Erectile Dysfunction.

Finally, 79.5% of the patients who initially referred DE had the diagnosis confirmed; the remaining were found to have low sexual desire (12.3%) or ED (8.2%) (Figure 4).

## 4. Discussion

This real-life study shows that a high percentage of patients attending an andrological outpatient clinic had a sexual dysfunction different from that they perceived and referred at the first visit. Moreover, during the long period of observation of this study, various other conditions were encountered, many of which do not have an official nosography.

Among the patients referring ED as an initial complain, 68.5% had this diagnosis confirmed, thus leaving about one third of patients affected by another dysfunction mistakenly perceived as ED. This misleading discrepancy was likely due to prejudice, false myths on male sexual performance, disinformation or terminology misunderstanding. In particular, 8.5% of the patients who apparently had ED were found to have decreased penile rigidity, not classifiable as ED, according to the official definition [24], because the degree of rigidity allowed them to penetrate, although the patient (and frequently the partner) perceived a lower degree of penile stiffness. This condition, typically subclinical, is referred to as “SED” [21] and can be bona fide considered a reason of psychological discomfort during sexual intercourse and beyond. Moreover, we observed decreased spontaneous erections, and an increased threshold to stimuli, which, in the previous sexual life of the patients, were sufficient to trigger erection (including audio-visual content, female voice through phone, unintentional physical contact, etc.). Psychological distress and somatization often underlie these subclinical conditions: despite few studies having investigated the relationship between somatoform symptoms and sexual function, this relationship has a severe impact on sexuality. [25].

The clinical approach to SED is still to be defined, as only few studies have evaluated non-ED conditions. A recent 15-year observational study conducted in Japanese men showed an association between penile rigidity decrement in each decade of life (40 s, 50 s, 60 s, and 70 s) and dissatisfaction with their own sexual function (except from men 70 years and above); this association was shown whether or not patients met the criteria for classical ED [26]. An objective quantification of penile rigidity (thus to find even slight differences in rigidity among the spectrum of non-ED patients) could be obtained through imaging techniques, such as virtual touch tissue quantification (VTTQ). This has been used to give numerical measurements of penile rigidity and can effectively and sensitively indicate the axial and radial rigidity changes in penile erection. VTTQ use has still to be defined in the clinical practice [27].

Interestingly, among the patients who required consultation for low sexual desire, only 57.5% had this diagnosis confirmed; 23% had ED (the most common misunderstanding in terminology was “loss of stimulation”) and 18.5% experienced an increased threshold to stimuli. Besides data from patients who underwent prostatectomy, only few studies have explored possible penile innervation alterations in para-physiologic conditions. These include rat models, which showed a pro-inflammatory role of dorsal penile block [28] and a correlation found between caloric restriction and age-related alterations of the cavernous neurovascular structure [29]. Another study reported a 40% prevalence of neuropathy of the dorsal nerve of the penis in a cohort of 44 patients complaining of penile sensory dysfunction, without correlation to major well known conditions, such as diabetes mellitus, myelopathy or prostatectomy [30]. A recent study [31] evaluated penile vibratory sensitivity measured through the penile sensitivity ratio (PSR), finding correlations of this parameter with age, diabetes and Peyronie’s disease. These studies suggest that an increased threshold to penile stimulation could be underestimated and requires greater attention by the clinician.

Among patients complaining of PE, the majority of them referred to have APE at the first visit. However, the final diagnosis frequently showed the opposite; the majority of them had LLPE which became worse over the years. Indeed, careful clinical investigation and the watchful patient’s interview showed a lack of control since the beginning of their sexual life, which was compensated by subsequent sexual intercourse. Hence, the patient realized the dysfunction only after experiencing the incapability to compensate with a second coitus with aging. This failure could be due to a worsening of anxiety or to age-related decrease of sexual endurance. These findings support the hypothesis that other unknown mechanisms may intervene in the elongation of IELT frequently experienced during subsequent coitus, when it is possible. The cerebro-spinal control of ejaculation consists of different arcs, some of which involve stimuli from the periphery to the cerebral cortex, while others resolve in lower circuits within the peripheral-spinal area [32,33]. We could hypothesize that if, during the second coitus, a temporary anesthetization of reflex arcs occurs, IELT may rise up. This mechanism could take place either in lower arcs (i.e., a refractoriness of penile dorsal nerve which stimulates interneuron in L3–L4 area) or in upper ones. In the latter possibility, minor stimuli from somatosensory inputs may result in a central effect of a partial “exhaustion” of the sexual drive due to the previous coitus. Moreover, ejaculation is closely associated with orgasm, a rewarding event where opioids are thought to play a role. The possible rise in endogenous opioids due to the first orgasm may thus be hypothesized as a further mechanism explaining how the first intercourse may influence the ability to control ejaculation in the next one. Binding the opioid receptors is, in fact, thought to be a mechanism increasing the threshold of excitation and opiatergic drugs have been studied in PE with some success [34,35]. If the aforementioned mechanisms, which, in animals, interact with both the dopaminergic and serotoninergic pathways, were proven, the central, serotoninergic etiology of LL-PE should be revised and integrated into a more complex model [36,37,38].

An IELT increase during the second coitus was similarly observed in the 6% of patients seeking consultation for ED who were not able to have a second intercourse because of the elongation of the refractory period. All these patients were found to have a PE, which the patient did not recognize as a problem until he was able to have a subsequent second sexual intercourse. The inability to compensate led the patients to ask for andrological help and further empathize the strict interplay between the ejaculatory and erection controls [39].

Patients who sought counseling for a lengthening of the refractory period were mostly found to have LLPE or difficulty in obtaining their partner’s orgasm. These data suggest that the second sexual intercourse is not simply due to male hyperactive desire, but it could be a strategy to compensate for PE (absolute compensation) or to cope with delayed female orgasm (relative compensation).

The diagnosis of DE was confirmed in the majority of patients seeking help for prolonged ejaculatory period (79.5% of patients), while 12.3% showed a low sexual desire and 8.2% an ED; thus, DE was the most reliable diagnosis at first consultation among all the conditions explored [12].

The assessment of sexual life quality is commonly explored using questionnaires available for clinical practice, such as the IIEF-15 [13]. IIEF-15 questions can be split into five domains: (a) erectile function, (b) orgasmic function, (c) sexual desire, (d) intercourse satisfaction, and overall satisfaction. Although IIEF-15 has been used as a valid diagnostic tool to assess the severity of ED, some “unmet needs” cannot be easily identified. In particular, IIEF-15 fails to identify (*i*) SED (not precluding the sexual intercourse), as the questions do not discern between grades of rigidity which still allow penetration; (*ii*) a prolonged refractory period, as the orgasmic function domain lacks a specific question related to the capability of reaching orgasm during the valid erection time; moreover, no questions relate to the loss of erection due to prolonged intercourse subsequent to prolonged refractory period; (*iii*) increased threshold to stimuli, as no questions explore penile sensitivity (the satisfaction explored by question #7 may be easily confused with other aspects of sexual intercourse, while the Orgasmometer [40] specifically measures orgasmic sensitivity); (*iv*) decreased frequency of spontaneous erections; (*v*) delayed orgasm in the female partner, or (*vi*) soft glans, as no questions assess these. The SIEDY questionnaire [41] may help to evaluate unmet needs, as specific questions relate to lower penile rigidity (not precluding the sexual intercourse), decreased frequency of spontaneous erections or delayed orgasm in the female partner. Anyway, neither IIEF-15 nor SIEDY are able to deepen the other conditions (prolonged refractory period, increased threshold to stimuli, soft glans), thus, the clinician has to ask specific questions exploring them.

Our data stress the need for clinicians to be aware of the spectrum of conditions that are not included in the official nosography, the “unmet needs” so frequently found in our retrospective survey, but still cause “sexual discomfort” and lead the patient to look for medical (in the best conditions) or non-medical (in the unhealthiest behaviors) help.

A limitation to our observation is the lack of data on the psychological background of the patients included in this study. Indeed, the presence of unexplored issues, such as conflicts within the family or within the couple, can represent a relevant factor contributing to patients’ own perceptions of the problem, as well as it contributing to sexual male dysfunction. Two observational studies reported a high association between family and/or couple conflicts and psychological comorbidities (i.e., anxiety, depression, and a worse peak systolic velocity measured through penile echo Doppler) [42] and between relational factors and several sexual dysfunctions (ED, PE, HSDD and reduced frequency of intercourses) [43] in patients consulting an andrology clinic for sexual dysfunction, respectively. A patient could describe a sexual dysfunction in different ways. Indeed, depending on the patient’s care for the couple’s sexual health, he can consider his IELT normal or not, according to the grade of concern for his ability to generate the partner’s orgasm during penetration.

Other limitations are the retrospective nature and the single-center design of our data, only partially reduced by the real-life quality and by the large number of patients treated in a homogeneous context.

Finally, another limitation is given by the lack of objectification of one of the final diagnoses, which is increased threshold to stimuli. The raise of penile stimulus needed to trigger erection is a parameter which is hard to quantify and exposed to psychological individual biases. As stated before, few studies have explored this issue.

The age of the patients seeking andrological help could be a major diagnosis determinant factor. It could be hypothesized that andrologists are culturally more prone to ask for sexological consultation in younger patients and to search only for organic etiologies in the older ones. This hypothesis could result in denying sexological help to older patients, which, however, is of paramount importance in the early diagnostic process [44]. This bias may come from the clinician’s misbelief that an organic cause is generally more “important” than a psychological one. In future studies, it will be interesting to evaluate the results according to the stratification of the patients for different age groups.

In conclusions, this study shows that classic nosography is often insufficient to define all male sexual disorders. Moreover, the frequent incapability, inability or impossibility of patients to communicate their innermost sexual and emotional experiences, due to prejudices, ignorance and misbeliefs as well to professional ineptitudes of the caregivers, lead to misunderstandings that potentially pave the way to a wrong diagnosis.

## Figures and Tables

**Table 1:** Prevalence of the symptoms that brought the patients to look for andrological consultation.

Type of Sexual Dysfunction	Number of Patients	Percentage
Erectile Dysfunction	5831	52%
Premature Ejaculation (PE)	3142	28.1%
• Life-Long PE	1017	9.1%
• Acquired PE	2125	19%
Low Sexual Desire	1284	11.5%
Delayed Ejaculation	444	4%
Others (Low Ejaculate Volume, Dysmorphophobia, Decreased Ejaculatory Power, “Unmet Needs” *)	499	4.4%

* The “unmet needs” included: lower penile rigidity (although not precluding the sexual intercourse), prolonged refractory period, increased threshold to stimuli, decreased frequency of spontaneous erections, delayed orgasm in the female partner, soft glans.

**Table 2:** Prevalence of final diagnoses at the end of a complete clinical investigation.

Type of Sexual Dysfunction	Number of Patients	Percentage
Erectile Dysfunction	4557	40.7%
Life-long Premature Ejaculation	2161	19.3%
Low Sexual Desire	968	8.6%
Acquired Premature Ejaculation	809	7.3%
Increased Threshold to Stimuli	530	4.7%
Subclinical Erectile Dysfunction	496	4.4%
Delayed Female Orgasm	440	3.9%
Delayed Ejaculation	353	3.2%
Prolonged Refractory Period + Premature Ejaculation	350	3.1%
Reduced Spontaneous Erections	233	2.1%
Soft Glans	17	0.2%
Other Diagnoses	286	2.5%
